# 33‐year‐old HIV‐positive patient presenting with primary effusion lymphoma

**DOI:** 10.1002/ccr3.1856

**Published:** 2018-10-09

**Authors:** Ihtesham A. Qureshi, Syed Zul‐Farah, Luis Carbajal, Aymara Chang

**Affiliations:** ^1^ Neurology Department Texas Tech University Health Sciences Center El Paso Texas; ^2^ Department of Internal Medicine Texas Tech University Health Sciences Center El Paso Texas

**Keywords:** HIV, non‐Hodgkin’s lymphoma, primary effusion lymphoma

## Abstract

Our patient presenting with symptoms of shortness of breath and fever, noncompliant with antiretroviral therapy was found to have a rare HIV‐associated non‐Hodgkin's lymphoma that carries a dismal prognosis. Early recognition of this condition with prompt treatment may provide a marginal benefit to the patient's overall life expectancy.

A 33‐year‐old man with past medical history of HIV (noncompliant on antiretroviral treatment; CD4 <20, HIV RNA PCR: 164000) brought to emergency department with symptoms of cough, shortness of breath, and fever. He was hospitalized for further evaluation. On physical examination finding, he appeared cachectic with tachycardia and jugular venous distention. Chest examination includes the presence of bibasilar crackles. Skin shows the presence of scattered purple lesions on his chest (Figure 1). A skin punch biopsy of the purple lesion was performed and sent to pathology for analysis using immunohistochemical stain revealed positive for HHV8. As part of his evaluation, he underwent echocardiogram that revealed pericardial effusion with findings concerning for pericardial tamponade requiring emergent pericardiocentesis. The pericardial fluid cytology revealed a diagnosis of primary effusion lymphoma (PEL). Computed tomography of chest indicated the presence of moderate‐sized bilateral pleural effusion (more on the right) requiring thoracocentesis (Figure 2). Pleural fluid cytology also confirmed findings consistent with PEL. He was started on first cycle of CHOP (Cyclophosphamide, Doxorubicin, Vincristine, Prednisone) in combination with antiretroviral therapy; however, unfortunately, despite our best efforts, patient decided to leave the hospital against medical advice.

**Figure 1:**
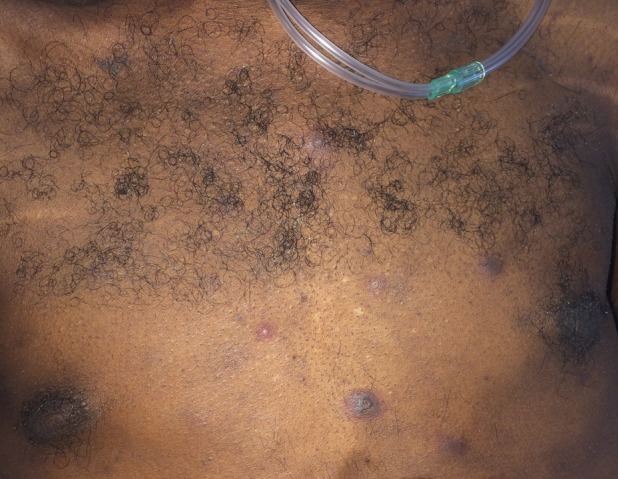
Picture depicting the presence of purple colored skin lesions suspicious for Kaposi's sarcoma confirmed on skin biopsy

**Figure 2:**
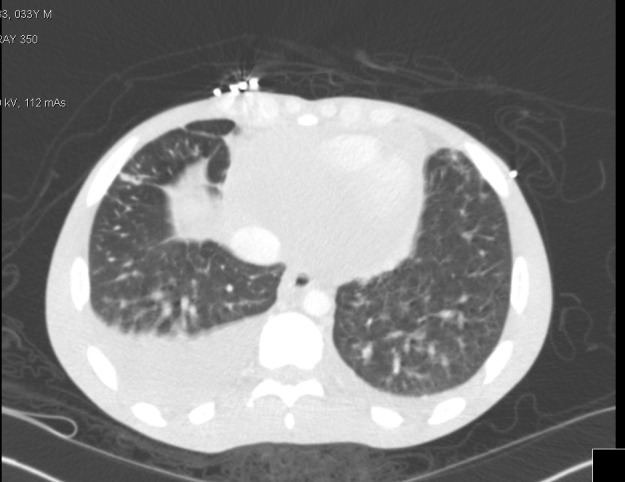
CT chest with contrast revealed the presence of peribronchovascular airspace consolidation in bilateral upper lobes with intrinsic cavities in association with bilateral pleural effusion (greater on the right)

Primary effusion lymphoma is considered a rare HIV‐related non‐Hodgkin's lymphoma (NHL) that constitutes for nearly 4% of all HIV‐related NHL.^1^ They have an increased tendency for arising within the body cavities (pleural space, pericardial, and peritoneum) is considered a unique clinical feature. Its presence reflects a dismal prognosis with an average survival time period of 6 months.^2^

## CONFLICT OF INTEREST

None.

## AUTHOR CONTRIBUTION

IAQ and ZF: involved in manuscript writing. LC: involved in the critical revision of the manuscript. AC: involved in patient care.
